# Cabozantinib Plus Atezolizumab or Cabozantinib Alone in Patients With Advanced NSCLC Previously Treated With an Immune Checkpoint Inhibitor: Results From the Phase 1b COSMIC-021 Study

**DOI:** 10.1016/j.jtocrr.2024.100666

**Published:** 2024-03-20

**Authors:** Joel W. Neal, Armando Santoro, Maria Gonzalez-Cao, Farah Louise Lim, Bruno Fang, Ryan D. Gentzler, Jerome Goldschmidt, Polina Khrizman, Claudia Proto, Shiven Patel, Sonam Puri, Stephen V. Liu, Erminia Massarelli, Denise Williamson, Martin Schwickart, Christian Scheffold, Svetlana Andrianova, Enriqueta Felip

**Affiliations:** a Division of Oncology, Stanford Cancer Institute, Palo Alto, California; b Department of Biomedical Sciences, Humanitas University, Pieve Emanuele, Milan, Italy; c IRCCS Humanitas Research Hospital- Humanitas Cancer Center, Rozzano, Milan, Italy; d Translational Cancer Research Unit, Instituto Oncologico Dr Rosell, Dexeus University Hospital, Barcelona, Spain; e Barts Health NHS Trust, St Bartholomew’s Hospital, London, United Kingdom; f Astera Cancer Care, East Brunswick, New Jersey; g Division of Hematology Oncology, University of Virginia Cancer Center, Charlottesville, Virginia; h Department of Medical Oncology, Blue Ridge Cancer Care, Blacksburg, Virginia; i MD Anderson Cancer Center at Cooper, Camden, New Jersey; j Medical Oncology Department, Fondazione IRCCS Istituto Nazionale Dei Tumori, Milan, Italy; k Division of Medical Oncology, The Huntsman Cancer Institute at the University of Utah, Salt Lake City, Utah; l Georgetown Lombardi Comprehensive Cancer Center, Washington, District of Columbia; m Department of Medical Oncology, City of Hope Comprehensive Cancer Center, Duarte, California; n Department of Biostatistics, Exelixis Inc, Alameda, California; o Department of Translational Medicine, Exelixis Inc, Alameda, California; p Department of Clinical Development, Exelixis Inc, Alameda, California; q Medical Oncology Service, Vall d’Hebron Institute of Oncology (VIHO), Vall d’Hebron Barcelona Hospital Campus, Universitat Autonoma de Barcelona, Barcelona, Spain

**Keywords:** Cabozantinib, Atezolizumab, Non–small cell lung cancer, Immunotherapy

## Abstract

**Introduction:**

We evaluated efficacy and safety of cabozantinib plus atezolizumab or cabozantinib alone in advanced NSCLC previously treated with an immune checkpoint inhibitor (ICI).

**Methods:**

COSMIC-021 (NCT03170960) is a phase 1b, multicenter study in advanced solid tumors. This analysis included patients with stage IV non-squamous NSCLC without actionable genomic aberrations in *EGFR, ALK, ROS1*, or *BRAF*-*V600E* who progressed on one prior ICI and less than or equal to two prior lines of systemic anticancer therapy. Patients received cabozantinib 40 mg orally/day plus atezolizumab 1200 mg intravenously every three weeks (combination cohort) or cabozantinib 60 mg orally/day (single-agent cabozantinib cohort). Primary end point of the combination cohort was objective response rate per Response Evaluation Criteria in Solid Tumors v1.1 by investigator. Outcomes in the single-agent cabozantinib cohort were exploratory.

**Results:**

Eighty-one patients assigned to combination therapy and 31 assigned to single-agent cabozantinib received greater than or equal to one dose of study treatment. Median (range) follow-up was 26.1 months (12.1–44.2) and 22.4 months (1.5–29.0), respectively. Objective response rate was 20% (95% confidence interval: 11.7%–30.1%) in combination cohort and 6% (95% confidence interval: 0.8%–21.4%) in single-agent cabozantinib cohort. Treatment-related adverse events (TRAEs) occurred in 86% of patients in the combination cohort and 90% in the single-agent cabozantinib cohort; grade 3/4 TRAEs were 44% and 48%, respectively. There were two grade 5 TRAEs: pneumonitis (n = 1, combination) and gastric ulcer hemorrhage (n = 1, single-agent). Neither PD-L1 expression in tumor cells nor tumor mutation burden correlated with outcomes.

**Conclusions:**

Cabozantinib plus atezolizumab demonstrated modest clinical activity and manageable toxicity in advanced NSCLC after progression on prior ICI.

## Introduction

First-line anti-PD-L1/PD-1 immune checkpoint inhibitors (anti-PD-(L)1 ICIs) with or without chemotherapy are standard of care for patients with advanced NSCLC without actionable genomic aberrations.^1^^,^^2^ Anti-PD-(L)1 ICIs are also the standard of care for patients with advanced NSCLC who progressed on platinum-based chemotherapy.^2^ However, despite advances in immunotherapy, the vast majority of patients with NSCLC experience disease progression,^3^, ^4^, ^5^, ^6^ and effective treatment options after progression on anti-PD-(L)1 ICIs are needed.^7^^,^^8^

Chemotherapy alone, or docetaxel with or without the VEGFR-targeting monoclonal antibody ramucirumab, are recommended as subsequent therapies in patients with NSCLC who previously received ICIs^1^^,^^2^; however, these standard-of-care therapies are associated with limited overall survival (OS) and high toxicity.^3^^,^^5^, ^6^, ^7^ In a phase 2 trial, the combination of ramucirumab with the PD-1 inhibitor pembrolizumab demonstrated a survival benefit versus standard of care in patients with advanced NSCLC after progression on prior ICIs.^7^ Therefore, the combination of a VEGF pathway–targeting agent with an ICI may help overcome resistance to ICIs.

Cabozantinib is a tyrosine kinase inhibitor (TKI) that targets multiple receptor tyrosine kinases, including MET, VEGFR, and the TAM family of kinases (TYRO3, AXL, MER), and has the potential to enhance response to ICIs.^9^ Cabozantinib in combination with ICIs has demonstrated efficacy in renal cell carcinoma and hepatocellular carcinoma in phase 3 studies.^10^^,^^11^ Atezolizumab is an anti-PD-L1 monoclonal antibody approved as monotherapy or in combination with chemotherapy for first-line treatment of advanced NSCLC with no *EGFR* or *ALK* genomic tumor aberrations.^6^ COSMIC-021 is a phase 1b study evaluating cabozantinib in combination with atezolizumab in multiple advanced solid tumors. Reported here are results in patients with advanced NSCLC who progressed following prior ICI therapy, treated with either cabozantinib plus atezolizumab (expansion combination cohort) or single-agent cabozantinib (exploratory single-agent cabozantinib cohort).

## Materials and Methods

### Study Design and Participants

COSMIC-021 is a multicenter, open-label, phase 1b study with a dose-escalation stage and a subsequent tumor-specific cohort expansion stage. The dose-escalation stage evaluated cabozantinib 40 mg and 60 mg in combination with atezolizumab and has been previously reported.^12^ The recommended dose of the combination for the cohort-expansion stage was cabozantinib 40 mg orally once daily plus atezolizumab 1200 mg intravenously every 3 weeks. Following the dose-escalation stage, patients were enrolled into tumor-specific cohorts in an expansion stage. Patients with NSCLC who were previously treated with an anti-PD-(L)1 ICI therapy were enrolled into two cohorts and treated with either cabozantinib plus atezolizumab (combination cohort) or single-agent cabozantinib (single-agent cabozantinib cohort).

For both cohorts, eligible patients were greater than or equal to 18 years of age with stage IV non-squamous NSCLC with radiographic progression on or after one prior anti-PD-(L)1 ICI therapy for metastatic disease. Patients were required to have measurable disease per Response Evaluation Criteria in Solid Tumors (RECIST) v1.1, Eastern Cooperative Oncology Group (ECOG) performance status of 0 or 1, adequate organ and marrow function, and availability of tumor tissue. Patients could have received up to two prior lines of systemic anticancer therapy to treat metastatic NSCLC (an anti–CTLA-4 agent was allowed; prior platinum-based chemotherapy was not required). Prior treatment with a VEGFR-targeting TKI was not allowed. Patients with tumors harboring known *EGFR*, *ALK*, *ROS1*, or *BRAF V600E* aberrations were excluded. Radiation therapy was not allowed within four weeks (two weeks for bone metastases) prior to study treatment initiation. Brain metastases must have been treated and stable for greater than or equal to four weeks before the first dose of study treatment. Patients with uncontrolled, significant intercurrent or recent illness were excluded.

This study adhered to the Good Clinical Practice guidelines and the Declaration of Helsinki. The study was approved by the institutional review board at each study center, and all patients provided written informed consent. This study was registered with ClinicalTrials.gov, NCT03170960.

### Procedures

Patients in the combination cohort received cabozantinib 40 mg orally once daily plus atezolizumab 1200 mg intravenously every three weeks, while patients in the single-agent cabozantinib cohort received single-agent cabozantinib 60 mg orally once daily; patients in the single-agent cabozantinib cohort who progressed on single-agent cabozantinib could cross over to receive the addition of atezolizumab. The first 51 patients were enrolled in a non-randomized fashion into the combination cohort to assess the clinical activity of the combination. Subsequent patients were randomized 1:1 to the combination or single-agent cohort using an unstratified permuted block design. Patients were treated until lack of clinical benefit, need for subsequent systemic anticancer treatment, or unacceptable toxicity. Treatment could be continued after radiographic progression if there was clinical benefit as determined by the investigator. Dose delays of cabozantinib and atezolizumab and dose reductions of cabozantinib (up to three reductions for single-agent cabozantinib cohort and two for combination cohort; 60 mg–40 mg daily, 40 mg–20 mg daily, 20 mg daily–20 mg every other day) were allowed to manage adverse events (AEs); dose reductions of atezolizumab were not allowed.

Tumor assessments by computed tomography or magnetic resonance imaging were performed per RECIST v1.1 by the investigator every six weeks for the first 12 months, then every 12 weeks thereafter.

Safety, including AE severity, relationship to study treatment, and relationship to immune effects, was assessed by the investigator according to the National Cancer Institute Common Terminology Criteria for Adverse Events v4.0.

Peripheral blood and tumor tissues were collected for exploratory biomarker analyses. Archival tumor tissue was obtained as formalin-fixed paraffin-embedded tumor blocks, or if unavailable, tumor slides. Fresh tumor tissue biopsies were optional. When available, blood samples were used to evaluate plasma, serum, and cellular biomarkers, and tumor tissue samples were used to evaluate changes in biomarker expression and genetic/genomic aberrations, but these assessments were not required. Tumor mutation burden (TMB), presence of genomic mutations (*KRAS*, *STK11*, *KEAP1,* or *STK11*), and PD-L1 expression in tumor cells were evaluated as follows for their effect on tumor response, OS, and progression-free survival (PFS). PD-L1 was assayed centrally (by Ventana SP263 assay, Roche Diagnostics, Florham Park, NJ) for patients with tumor samples available; other patients had PD-L1 status determined by the investigator or had unknown status. Formalin-fixed paraffin-embedded tumor tissue used in immunohistochemistry analyses and matched blood samples were used for whole exome sequencing to determine TMB. A DNA library was generated from fragmented genomic DNA using Agilent SureSelectXT Human all Exon 50 Mb v6 capture baits (Agilent, Santa Clara, CA) and sequenced by sequencing-by-synthesis technology (SBS, Illumina, San Diego, CA) with a read depth of 250× for tumor tissue and 50× for blood samples. TMB was calculated by excluding germline variants, variants with a maximum population frequency of greater than 0.1% in the 1000 genomes project (National Heart Lung and Blood Institute Grand Opportunity Exome Sequencing Project or gnomAD), and variants that failed the variant quality filter. Variant calls were restricted to include only variants located within high confidence regions of protein coding sequence.

### End Points and Assessments

The primary end point of the combination cohort was objective response rate (ORR; defined as the proportion of patients who achieved a confirmed partial or complete response as best response) per RECIST v1.1 by investigator. The secondary end point was safety through evaluation of AEs, serious AEs, and AEs of special interest (AESIs; immune-mediated AEs associated with ICIs, cases of potential drug-induced liver injury, and suspected transmission of an infectious agent by the study treatment). Exploratory end points included the following: duration of response (DOR; defined as the time between first observed tumor response and subsequent first occurrence of disease progression or death) and PFS (defined as time from first dose to the earlier of radiographic progression or death from any cause) by investigator per RECIST v1.1; ORR, DOR, and PFS by blinded independent radiology committee (BIRC) per RECIST v1.1; OS (defined as time from first dose to death from any cause); and biomarker analyses.

End points of the single-agent cabozantinib cohort were exploratory and included all efficacy and safety analyses conducted for the combination cohort (except subgroup analyses), and biomarker analyses.

### Statistical Analysis

All analyses were conducted in the safety population, which is defined as all patients who received any study treatment. Continuous data were summarized using descriptive statistics; categorical data were summarized using frequencies and percentages. ORR per RECIST v1.1 was evaluated with a two-sided 95% exact binomial confidence interval (CI), using the Clopper-Pearson method. Median DOR, PFS, OS, and associated 95% CIs were estimated using the Kaplan–Meier method. Exploratory modeling analyses including survival analysis with time-dependent covariates, general linear models, and mixed models with repeated measures were employed to assess relationships between biomarker measurements and ORR, PFS, and OS. Maximum percent tumor reduction from baseline in target lesions was assessed based on patients in the safety population. All analyses were conducted using SAS version 9.3 or higher (SAS Institute, Cary, NC).

## Results

### Patients

**Figure 1:**
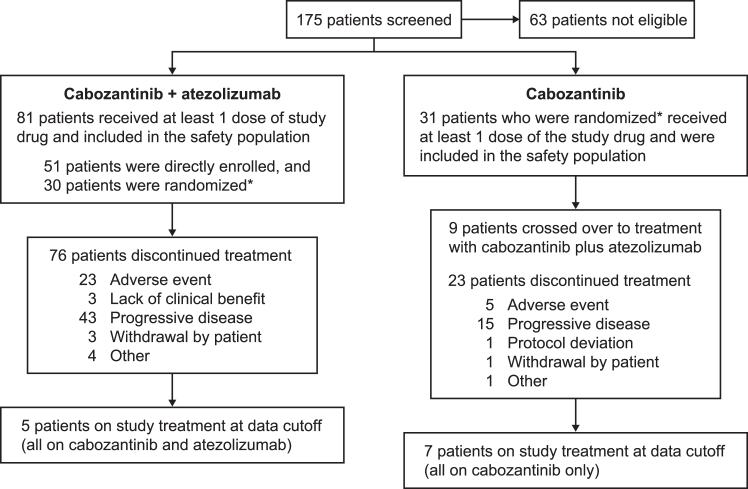
Patient disposition. ∗The first 51 patients were enrolled in a non-randomized fashion into the cabozantinib + atezolizumab cohort, and subsequent patients were randomized 1:1 between the cabozantinib + atezolizumab and cabozantinib cohorts.

Between April 2018 and December 2020, 112 patients with NSCLC who were enrolled in the combination cohort (n = 81) and the single-agent cabozantinib cohort (n = 31) received at least one dose of study treatment and were included in the safety population (Fig. 1). Median age (range) was 67 years (38–93) in the combination cohort and 70 years (48–92) in the single-agent cabozantinib cohort. Most patients had an ECOG performance status of 1 (64% [52 of 81] for the combination cohort; 71% [22 of 31] for the single-agent cabozantinib cohort; Table 1).

**Table 1:** Baseline Characteristics

Characteristic	Cabozantinib + Atezolizumab (n = 81)	Cabozantinib (n = 31)
Age, median (range), years	67 (38–93)	70 (48–92)
Male, n (%)	46 (57)	18 (58)
Race, n (%)		
Asian	1 (1)	0
Black/African American	3 (4)	1 (3)
White	71 (88)	27 (87)
Not reported	6 (7)	3 (10)
Geographic region, n (%)		
North America	38 (47)	14 (45)
Europe/Australia	43 (53)	17 (55)
Smoking history		
Current	14 (17)	3 (10)
Former	55 (68)	23 (74)
Never	12 (15)	5 (16)
ECOG performance status, n (%)		
0	28 (35)	9 (29)
1	52 (64)	22 (71)
2	1 (1)	0
Histology subtype, n (%)^a^		
Adenocarcinoma	76 (94)	31 (100)
Large cell carcinoma	2 (2)	0
Other	1 (1)	0
Sites of tumor, n (%)		
Lung	68 (84)	27 (87)
Lymph node	44 (54)	19 (61)
Bone	24 (30)	9 (29)
Liver	17 (21)	7 (23)
Adrenal	13 (16)	7 (23)
Brain	11 (14)	2 (6)
Kidney	4 (5)	1 (3)
Spleen	4 (5)	1 (3)
Other	2 (2)	0
Number of tumor sites per investigator per RECIST 1.1, n (%)		
1	16 (20)	5 (16)
2	18 (22)	11 (35)
≥3	47 (58)	15 (48)
PD-L1 status, n (%)^b^		
<1%	37 (46)	12 (39)
≥1%	40 (49)	19 (61)
1%–49%	18 (22)	9 (29)
≥50%	19 (23)	6 (19)
Unknown	4 (5)	0
Prior lines of systemic anticancer therapy, n (%)^c^		
1	35 (43)	14 (45)
2	45 (56)	14 (45)
≥3^d^	1 (1)	2 (6)
Prior selected systemic anticancer (non-radiation) therapies, n (%)^c^		
Prior anti-PD-(L)1 or CTLA-4 therapy	81 (100)	30 (97)
Prior chemotherapy	66 (81)	24 (77)
Platinum-based	65 (80)	24 (77)
Non-platinum-based	63 (78)	24 (77)
Prior anti-PD-(L)1 treatment, n (%)^c^		
Anti-PD-(L)1 alone	14 (17)	5 (16)
Anti-PD-(L)1 + non-platinum chemotherapy	1 (1)	0
Anti-PD-(L)1 + platinum-containing chemotherapy	65 (80)	24 (77)
Concurrent	29 (36)	11 (35)
Platinum-containing chemotherapy followed by anti- PD-L1/PD-1	31 (38)	11 (35)
Anti-PD-(L)1 ICI followed by platinum-containing chemotherapy	2 (2)	1 (3)
Best response to prior anti-PD-(L)1 ICI therapy, n (%)		
Complete response	1 (1)	0
Partial response	14 (17)	6 (19)
Stable disease	35 (43)	7 (23)
Progressive disease	22 (27)	13 (42)
Not evaluable	0	1 (3)
Unknown	9 (11)	4 (13)

CTLA-4, cytotoxic T-lymphocyte–associated antigen 4; ECOG, Eastern Cooperative Oncology Group; ICI, immune checkpoint inhibitor; PD-1, programmed death-1; PD-L1, programmed death-ligand 1; RECIST, Response Evaluation Criteria in Solid Tumors.

a Histology of two patients was missing in the combination arm.

b PD-L1 expression in tumor cells; based on central assessment using SP263 assay or local assessment per investigator.

c For locally advanced or metastatic disease.

d Three patients who reported having three lines of therapy were confirmed to have had 1 or 2 lines of therapy post data cutoff.

Within each cohort, nearly equal proportions of patients received one and two prior lines of systemic anticancer therapy for locally advanced or metastatic disease, 35 patients (43%) and 45 patients (56%) in the combination cohort and 14 patients (45%) and 14 patients (45%) in the single-agent cabozantinib cohort, respectively. Prior platinum-containing chemotherapy was received by 65 patients (80%) in the combination cohort and 24 patients (77%) in the single-agent cabozantinib cohort. Prior anti-PD-(L)1 ICI treatment was received without prior chemotherapy by 14 patients (17%) in the combination cohort and five patients (16%) in the single-agent cabozantinib cohort; concurrently with platinum-containing chemotherapy by 29 patients (36%) and 11 patients (35%); following platinum-containing chemotherapy by 31 patients (38%) and 11 patients (35%); and prior to platinum-containing chemotherapy by two patients (2%) and one patient (3%). In the combination cohort, the best response to prior anti-PD-(L)1 ICI therapy was complete response in one patient (1%), partial response in 14 patients (17%), stable disease in 35 patients (43%), and progressive disease in 22 patients (27%); in the single-agent cabozantinib cohort, the best response to prior anti-PD-(L)1 ICI therapy was partial response in six patients (19%), stable disease in seven patients (23%), and progressive disease in 13 patients (42%).

The median (range) duration of follow-up was 26.1 months (12.1–44.2) in the combination cohort and 22.4 months (1.5–29.0) in the single-agent cabozantinib cohort. At the data cutoff, five of 81 patients (6%) in the combination cohort and seven of 31 patients (26%) in the single-agent cabozantinib cohort remained on study treatment. The most common reason for treatment discontinuation was progressive disease in both cohorts (53% [43 of 81] in combination cohort and 48% [15 of 31] in single-agent cabozantinib cohort; Fig. 1). Subsequent therapy was received by 24 patients (30%) in the combination cohort and six (19%) in the single-agent cabozantinib cohort (not including patients who crossed over to cabozantinib plus atezolizumab; Supplementary Table 1).

### Efficacy

ORR per RECIST v1.1 by investigator was 20% (16/81; 95% CI: 11.7%–30.1%) in the combination cohort and 6% (2/31; 95% CI: 0.8%–21.4%) in the single-agent cabozantinib cohort (Table 2); all responses in both cohorts were confirmed partial responses. The disease control rate (DCR; best overall response of complete response + partial response + stable disease greater than or equal to 12 weeks) was 80% (65/81; 95% CI: 69.9%–88.3%) for the combination cohort and 65% (20/31; 95% CI: 45.4%–80.8%) for the single-agent cabozantinib cohort; and 16% (13 of 81) and 19% (6 of 31), respectively, had a best response of progressive disease. When assessed by BIRC, ORR was 10% (8 of 81) in the combination cohort and 6% (2 of 31) in the single-agent cabozantinib cohort (Supplementary Table 2). In the combination cohort, responses were observed regardless of ECOG Performance Status (PS) score, PD-L1 status, response to prior single-agent ICI or ICI-containing regimen, or most recent therapy including ICI or not (Supplementary Table 3). Among patients with known PD-L1 status, response rates by investigator were numerically higher in those with PD-L1 positive tumors (PD-L1 ≥1%), and highest in patients with PD-L1 greater than or equal to 50%. DCR was similar across all subgroups (ECOG PS, PD-L1 status, response to prior single-agent ICI or ICI-containing regimen, and most recent therapy including ICI or not).

**Table 2:** Tumor Response per RECIST v1.1 by Investigator

Response Parameter	Cabozantinib + Atezolizumab (n = 81)	Cabozantinib (n = 31)
ORR, % (95% CI)	20 (11.7–30.1)	6 (0.8–21.4)
Best overall response, n (%)		
Confirmed complete response	0	0
Confirmed partial response	16 (20)	2 (6)
Stable disease	49 (60)	18 (58)
Progressive disease	13 (16)	6 (19)
Missing	3 (4)	5 (16)
Disease control rate, % (95% CI)	80 (69.9–88.3)	65 (45.4–80.8)
Duration of response, median (95% CI), months	5.8 (4.2–6.9)	10.6 (6.3–NE)

*Note:* Disease control rate = complete response + partial response + stable disease ≥ 12 weeks. CI, confidence interval; NE, not estimable; ORR, objective response rate; RECIST, Response Evaluation Criteria in Solid Tumors.

**Figure 2:**
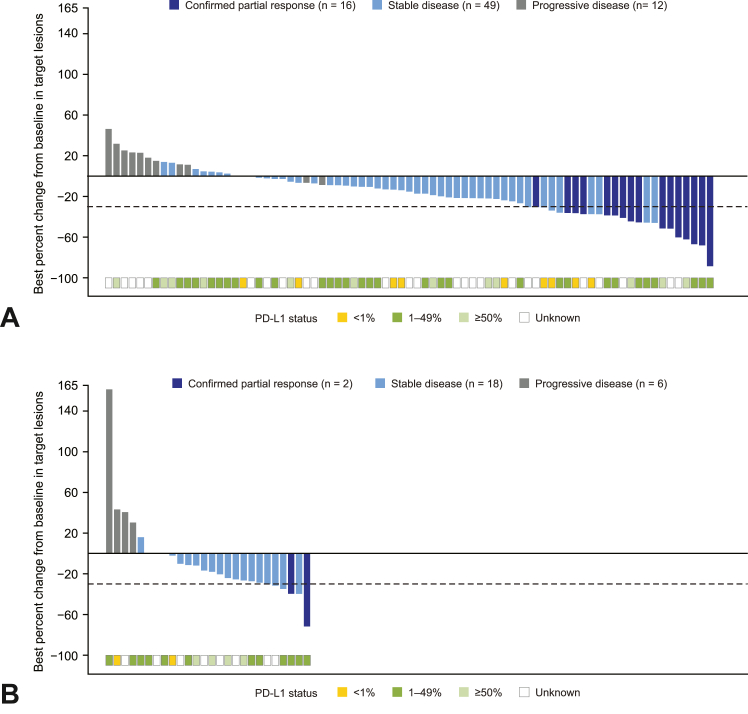
Waterfall plot for maximum percent change from baseline in target lesions per RECIST v1.1 by investigator in (*A*) combination cohort and (*B*) single-agent cabozantinib cohort. Maximum percentage of reduction or minimum increase from baseline in sum of diameters of target lesions before progressive disease or initiation of any non-protocol anticancer therapy. Only patients with at least one baseline and post-baseline radiographic tumor assessment are shown. Any reduction in the sum of diameter in target lesion was observed in 72% (58 of 81) of patients in the combination cohort and 58% (18 of 31) of patients in the single-agent cabozantinib cohort. PD-L1, programmed death-ligand 1; RECIST, Response Evaluation Criteria in Solid Tumors.

Median DOR per RECIST v1.1 by investigator was 5.8 months (95% CI: 4.2–6.9) in the combination cohort (Table 2). DOR in the combination cohort was similar across subgroups by ECOG PS, PD-L1 status, and response to prior ICI (Supplementary Table 3). Seventy-two percent (58 of 81) of evaluable patients in the combination cohort and 58% (18 of 31) of evaluable patients in the single-agent cabozantinib cohort had any reduction in target lesions from baseline (Fig. 2). Change in sum of target lesions over time per RECIST v1.1 by investigator is shown in Supplementary Figure 1. Of the patients in the combination cohort remaining on cabozantinib plus atezolizumab treatment at data cutoff, three had an ongoing partial response and one of these patients had been treated for more than 27 months (Supplementary Figure 2). In the single-agent cabozantinib cohort, both patients who achieved a partial response with cabozantinib had discontinued treatment (one patient at ∼10 months, and the other at ∼16 months).

**Figure 3:**
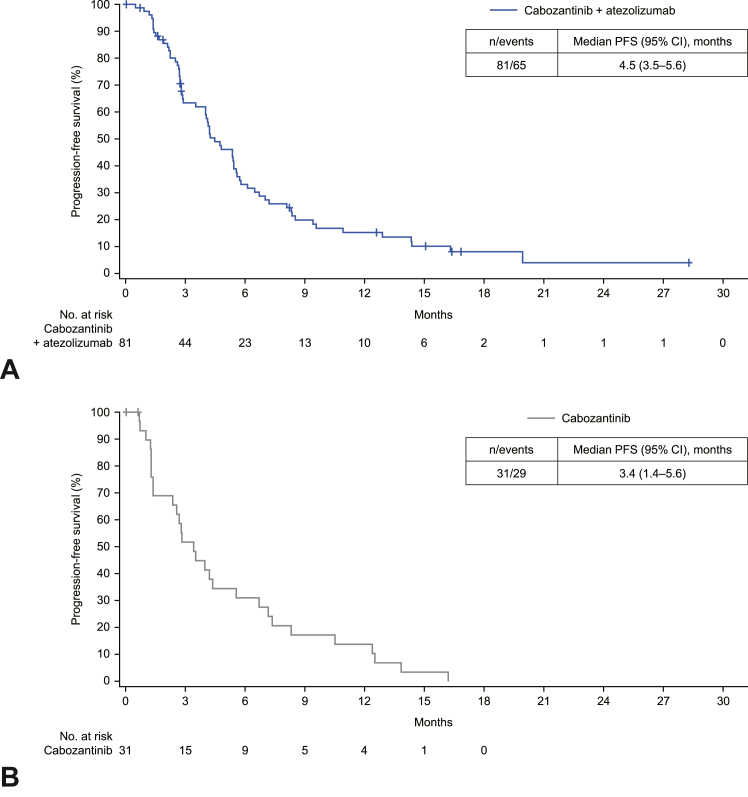
Progression-free survival per RECIST v1.1 by investigator in (*A*) combination cohort and (*B*) single-agent cabozantinib cohort. CI, confidence interval; No., number; PFS, progression-free survival; RECIST, Response Evaluation Criteria in Solid Tumors.

The median PFS per RECIST v1.1 by investigator was 4.5 months (95% CI: 3.5–5.6) in the combination cohort and 3.4 months (95% CI: 1.4–5.6) in the single-agent cabozantinib cohort (Fig. 3); median PFS by BIRC was 4.7 months (95% CI: 4.0–5.6) and 3.5 months (95% CI: 2.3–6.7), respectively. PFS estimates by investigator at 6 and 12 months, respectively, were 33% (95% CI: 23%–44%) and 15% (95% CI: 8%–25%) for combination cohort and 31% (95% CI: 16%–48%) and 14% (95% CI: 4%–29%) for single-agent cabozantinib cohort. Median PFS in subgroups were similar to that of the overall combination cohort population (Supplementary Table 3).

The median OS was 12.8 months (95% CI: 7.2–15.7) for combination cohort and 8.1 months (95% CI: 4.5–11.5) for single-agent cabozantinib cohort (Supplementary Fig. 3); OS estimates at 6 and 12 months, respectively, were 68% (95% CI: 56%–77%) and 51% (95% CI: 40%–62%) for combination cohort and 58% (95% CI: 39%–73%) and 29% (95% CI: 15%–45%) for single-agent cabozantinib cohort. In the combination cohort, median OS was similar regardless of PD-L1 status. Patients with an ECOG PS of 0 had a higher median OS (16.3 months [95% CI: 10.0–26.9]) compared with those with ECOG PS of 1 (9.6 months [95% CI: 6.0– 14.5]; Supplementary Table 3).

### Safety

At the data cutoff, median (range) duration of study treatment exposure was 5.2 months (0.3–30.2) in the combination cohort and 3.5 months (0.7–16.4) in the single-agent cabozantinib cohort (Supplementary Table 4). Treatment-emergent AEs occurred in all patients in each cohort (Supplementary Table 5) and led to dose modification of cabozantinib (delays or dose reductions) in 77% of patients in the combination cohort and 87% in the single-agent cabozantinib cohort. Treatment-emergent AEs led to delay of atezolizumab in 33% in the combination cohort.

Treatment-related AEs (TRAEs) occurred in 86% of patients in the combination cohort and 90% of patients in the single-agent cabozantinib cohort (Table 3). The most common of these included diarrhea (40% in combination cohort and 42% in single-agent cabozantinib cohort), fatigue (28% and 19%), decreased appetite (25% and 23%), nausea (22% and 42%), and asthenia (22% and 32%). Grade 3/4 TRAEs occurred in 44% in the combination cohort and 48% in the single-agent cabozantinib cohort. The most frequent included hypertension (4% in combination cohort and 19% in single-agent cabozantinib cohort) and asthenia (4% and 6%). TRAEs leading to discontinuation of cabozantinib were reported in 15% in the combination cohort and 6% in the single-agent cabozantinib cohort; those leading to discontinuation of atezolizumab were reported in 11% in the combination cohort. There was one treatment-related grade 5 event in each cohort: pneumonitis in combination cohort and gastric ulcer hemorrhage (patient was receiving concomitant anticoagulation) in the single-agent cabozantinib cohort.

**Table 3:** Treatment-Related Adverse Events

TRAE	Cabozantinib + Atezolizumab (n = 81)		Cabozantinib (n = 31)	
	Any grade	Grade 3/4	Any grade	Grade 3/4
Any event, n (%)	70 (86)	36 (44)	28 (90)	15 (48)
Diarrhea	32 (40)	1 (1)	13 (42)	1 (3)
Fatigue	23 (28)	3 (4)	6 (19)	0
Decreased appetite	20 (25)	0	7 (23)	1 (3)
Asthenia	18 (22)	3 (4)	10 (32)	2 (6)
Nausea	18 (22)	1 (1)	13 (42)	2 (6)
Palmar-plantar erythrodysesthesia	17 (21)	3 (4)	4 (13)	0
Aspartate aminotransferase increased	14 (17)	1 (1)	9 (29)	0
Hypertension	14 (17)	3 (4)	9 (29)	6 (19)
Alanine aminotransferase increased	13 (16)	1 (1)	7 (23)	1 (3)
Vomiting	11 (14)	0	7 (23)	1 (3)
Proteinuria	10 (12)	3 (4)	3 (10)	0
Hypomagnesemia	9 (11)	1 (1)	2 (6)	0
Hypothyroidism	9 (11)	0	4 (13)	0
Anemia	8 (10)	2 (2)	4 (13)	1 (3)
Dysgeusia	7 (9)	0	5 (16)	0
Stomatitis	6 (7)	0	6 (19)	0
Thrombocytopenia	2 (2)	0	6 (19)	0

*Note:* This table reports treatment-related AEs that occurred in ≥10% of patients. There was one grade 5 treatment-related event of pneumonitis in the combination cohort and one of gastric ulcer hemorrhage in the single-agent cabozantinib cohort.

AESIs occurred in 73% of patients in the combination cohort and 71% in the single-agent cabozantinib cohort (Supplementary Table 6). The most frequent AESIs were rash (42% in combination cohort and 35% in single-agent cabozantinib cohort), hepatitis (lab abnormalities and diagnosis; 35% and 45%), pancreatitis (17% and 6%) and hypothyroidism (16% and 23%). Grade 3/4 AESIs occurred in 26% in the combination cohort and 10% in the single-agent cabozantinib cohort. High dose steroids (≥40 mg of prednisone daily or equivalent) were required for AEs in 20 patients (25%) in the combination cohort and in seven (23%) in the single-agent cabozantinib cohort.

### Biomarkers

There was no significant correlation between any of the assessed biomarkers (TMB, PD-L1 expression in tumor cells, and *KRAS*, *STK11*, or *KEAP1/STK11* mutations) and tumor response, OS, or PFS. PD-L1 data are shown in Supplementary Table 3, and other biomarker data are shown in Supplementary Figures 4 and 5.

## Discussion

In the combination cohort of the phase 1b COSMIC-021 study, cabozantinib plus atezolizumab demonstrated encouraging clinical activity in patients with non-squamous NSCLC who had progressed following prior ICI therapy, with an ORR of 20% when assessed by investigator (primary end point) and 10% when assessed by BIRC. Modest clinical activity was observed with single-agent cabozantinib (single-agent cabozantinib cohort), with an ORR of 6%. Median PFS and OS were numerically longer with cabozantinib plus atezolizumab compared with single-agent cabozantinib, suggesting that rechallenge with immunotherapy may provide some clinical benefit. The safety profile of cabozantinib plus atezolizumab was consistent with the known safety profiles for the individual treatment components, and AEs were manageable with dose modification.

The combination and single-agent cabozantinib cohorts of the COSMIC-021 study comprised a NSCLC patient population in need of novel treatments after progression on immunotherapy. Single-agent chemotherapy (including docetaxel) and the combination of ramucirumab plus docetaxel are standard-of-care treatment options for these patients who progressed on immunotherapy.^2^ However, there is lack of clinical data on the efficacy and safety of these therapies following progression on ICIs, and these treatments have shown limited efficacy and high toxicity in the second-line setting. In a phase 3 study in patients with advanced NSCLC who progressed after first-line platinum-based chemotherapy, median OS was 10.5 months with ramucirumab plus docetaxel and 9.1 months with docetaxel alone; grade greater than or equal to 3 AEs occurred in 70% and 71%, respectively.^13^ Real-world studies have reported response rates of up to 60% with ramucirumab plus docetaxel post-ICI therapy, but the treatment was associated with a high frequency of AEs.^8^^,^^14^

Combination strategies may improve the efficacy of immunotherapy, including combinations with TKIs, which can promote an immune-permissive tumor microenvironment and avoid the toxicity associated with chemotherapy. In COSMIC-021, cabozantinib plus atezolizumab demonstrated encouraging clinical activity, including objective responses in patients who had stable or progressive disease as best response to their prior ICI treatment, suggesting that the addition of a TKI may facilitate resensitization to ICIs. In this study, no clear association was found between biomarkers assessed (TMB, PD-L1 status, presence of *KRAS*, *STK11*, or *KEAP1/STK11* mutations) and response, PFS, or OS, indicating that the ICI plus TKI combination may benefit a heterogeneous patient population.

In addition to cabozantinib plus atezolizumab, other vascular endothelial growth factor pathway agents have been evaluated in combination with ICIs following prior immunotherapy. The phase 2 Lung-MAP S1800A study evaluated ramucirumab in combination with pembrolizumab versus standards of care, including ramucirumab plus docetaxel and single-agent chemotherapy, in patients with advanced NSCLC who previously received immunotherapy.^7^ ORR, PFS, and OS with cabozantinib plus atezolizumab in COSMIC-021 were similar to those reported with ramucirumab plus pembrolizumab. However, it is important to note that differences in the patient population between the studies limit any comparisons of outcomes. Although the ramucirumab plus pembrolizumab study enrolled patients with acquired resistance to ICI within at least 84 days after ICI treatment initiation, our study did not select patients based on type of ICI resistance. In addition, patients in the ramucirumab plus pembrolizumab study had squamous or non-squamous NSCLC; our study included only patients with non-squamous NSCLC.

Based on encouraging clinical activity observed in the current analysis, cabozantinib plus atezolizumab was further evaluated in patients with NSCLC in the phase 3 CONTACT-01 study. Patients in CONTACT-01 had metastatic NSCLC that was non-squamous (75%) or squamous (25%) and had progressed after an anti-PD-(L)1 ICI therapy in combination with or subsequent to chemotherapy. The study did not meet its primary end point of OS with cabozantinib plus atezolizumab versus docetaxel (median 10.7 months for the combination versus 10.5 months for docetaxel).^15^ It is worth noting that differences in study populations limit comparisons between CONTACT-01 and COSMIC-021; COSMIC-021 only included patients with non-squamous NSCLC, and progression on prior platinum-containing chemotherapy was not an eligibility requirement (81% received prior chemotherapy).

One limitation of this analysis of COSMIC-021 was that it was not designed for direct comparison of cabozantinib plus atezolizumab versus single-agent cabozantinib, and different starting doses of cabozantinib were used in each cohort (40 mg in the combination cohort, 60 mg in the single-agent cabozantinib cohort). In addition, it is notable that the investigator-assessed ORR for combination therapy was double that of the BIRC-assessed rate, which is consistent with prior reports showing that investigators tend to overestimate the ORR compared with BIRC.^16^ In addition, the study was limited by sample size, and the cabozantinib plus atezolizumab cohort was comprised of non-randomized and randomized patients (patients were initially enrolled into the combination cohort to assess clinical activity, and subsequent patients were randomized to the combination cohort or single-agent cabozantinib cohort). Biomarker analyses were also limited by the small number of patients in each analysis.

Other phase 3 studies have also evaluated the role of TKIs in combination with ICIs versus standard-of-care chemotherapy in patients with NSCLC who progressed on prior immunotherapy (LEAP-008 [NCT03976375]; SAPPHIRE [NCT03906071]).^17^^,^^18^ These studies did not meet their primary end point of improving OS.^17^^,^^18^ More detailed information from these studies may help with the design of future clinical trials in this difficult-to-treat patient population.

In conclusion, cabozantinib plus atezolizumab demonstrated modest clinical activity in patients with advanced non-squamous NSCLC who progressed on prior anti-PD-(L)1 ICI therapy. Clinical activity was observed regardless of ECOG PS, prior response to ICIs, or PD-L1 status. Safety was consistent with the known safety profiles of the individual agents. None of the evaluated biomarkers were associated with outcomes. There remains an unmet need to improve on the longstanding standard of cytotoxic chemotherapy in the treatment of NSCLC that is refractory to immunotherapy and platinum-based chemotherapy.

## CRediT Authorship Contribution Statement

**Joel W. Neal:** Conceptualization, Visualization, Investigation, Supervision, Writing - original draft, Writing - review and editing.

**Armando Santoro:** Investigation, Supervision, Writing - review & editing.

**Maria Gonzalez-Cao:** Investigation, Supervision, Writing - review & editing.

**Farah Louise Lim:** Investigation, Supervision, Writing - review & editing.

**Bruno Fang:** Investigation, Supervision, Writing - review & editing.

**Ryan D. Gentzler:** Investigation, Supervision, Writing - review & editing.

**Jerome Goldschmidt:** Investigation, Supervision, Writing - review & editing.

**Polina Khrizman:** Investigation, Supervision, Writing - review & editing.

**Claudia Proto:** Investigation, Supervision, Writing - review & editing.

**Shiven Patel:** Investigation, Supervision, Writing - review & editing.

**Sonam Puri:** Investigation, Supervision, Writing - review & editing.

**Stephen V. Liu:** Investigation, Supervision, Writing - review & editing.

**Erminia Massarelli:** Investigation, Supervision, Writing - review & editing.

**Enriqueta Felip:** Investigation, Supervision, Writing - review & editing.

**Denise Williamson:** Data curation, Formal analysis, Resources, Writing - review & editing.

**Martin Schwickart:** Data curation, Formal analysis, Resources, Writing - review & editing.

**Christian Scheffold:** Data curation, Formal analysis, Resources, Writing - review & editing.

**Svetlana Andrianova:** Data curation, Formal analysis, Resources, Writing - review & editing.

## Disclosure

Dr. Neal reported receiving honoraria from Biomedical Learning Institute CME, Clinical Care Options, CME Matter, Medscape CME, MJH Life Sciences CME, MLI Peerview CME, Prime Oncology CME, Projects in Knowledge CME, Research to Practice CME, Rockpointe CME, Medical Educator Consortium, HMP Education, a consulting or advisory role with AstraZeneca, Blueprint Pharmaceuticals, Calithera Biosciences, D2G Oncology, Exelixis, Genentech, Roche, Jounce Therapeutics, Eli Lilly and Company, Natera, Regeneron, Sanofi, Surface Oncology, Takeda Pharmaceuticals, Turning Point Therapeutics, and receiving institutional research funding from 10.13039/100006483AbbVie, Adaptimmune, Boehringer Ingelheim, 10.13039/100010544Exelixis, 10.13039/100004328Genentech, 10.13039/100004337Roche, GSK, 10.13039/100005565Janssen, 10.13039/100004334Merck, Nektar Therapeutics, 10.13039/100004336Novartis, Takeda Pharmaceuticals. Dr. Armando Santoro reported having a consulting or advisory role with Sanofi, Incyte, receiving honoraria from AbbVie, Amgen, ArQule, AstraZeneca, Bayer, BMS, Celgene, Eisai, Gilead Sciences, Lilly, MSD, Novartis, Pfizer, Roche, Sandoz, Servier, Takeda, and participating in a data safety or advisory board with BMS, Servier, Gilead, Pfizer, Eisai, Bayer, MSD. Dr. Maria Gonzalez-Cao reported receiving grants or contracts from Roche, AstraZeneca, and Novartis, honoraria from Bristol Myers Squibb, MSD, AstraZeneca, Novartis, and Pierre Fabre, support for travel, accommodations, and expenses from MSD, AstraZeneca, and Pierre Fabre, and having a leadership or fiduciary role with the Spanish Melanoma Group. Dr. Ryan D. Gentzler reported receiving honoraria from Targeted Oncology, OncLive, Clinical Care Options, Society for Immunotherapy of Cancer, American Society of Clinical Oncology, MedStar Health, Aptitude Health, support for attending meetings from International Association for the Study of Lung Cancer, American Society of Clinical Oncology, Dava Oncology, Tempus, having a consulting or advisory role with AstraZeneca, Daiichi Sankyo, Gilead, Janssen, Jazz Pharmaceuticals, Mirati Therapeutics, OncoCyte, Sanofi, Takeda, Merus, Regeneron, receiving institutional research funding from 10.13039/100002429Amgen, Alliance Foundation, 10.13039/100004325AstraZeneca, Big Ten Research Consortium, 10.13039/100002491Bristol Myers Squibb, 10.13039/100010795Chugai, 10.13039/501100022274Daiichi Sankyo, 10.13039/100008130Helsinn Therapeutics, Hoosier Cancer Research Network, 10.13039/100005565Janssen, 10.13039/100016765Jounce Therapeutics, Merck, 10.13039/100016957Mirati Therapeutics, 10.13039/100004319Pfizer, 10.13039/100008199RTI International, Takeda, Dizal, Puma, and having leadership or committee roles with Hoosier Cancer Research Network, ASCO, Journal of Clinical Oncology, NCI Investigational Drug Steering Committee, and International Association for the Study of Lung Cancer. Dr. Jerome Goldschmidt reported receiving honoraria from G1 Therapeutics, and support for travel, accommodations, and expenses from Sara Cannon Research Institute. Dr. Claudia Proto reports receiving honoraria from AstraZeneca, Bristol Myers Squibb, MSD, Roche, Sanofi, having a consulting or advisory role with AstraZeneca, MSD and Roche, institutional research funding from MSD, Lilly, Pfizer, support for travel, accommodations, expenses from AstraZeneca, MSD, and Roche, and is the principal investigator in clinical trials for Janssen, Pfizer, Lilly, Spectrum Pharmaceuticals, Roche, MSD, BMS, and AstraZeneca. Dr. Shiven Patel reported receiving a consulting or advisory role with AstraZeneca, Blueprint Medicines, Boehringer Ingelheim, Regeneron, Sanofi, TerSera Therapeutics LLC, Total Health Conferencing, Natera, Takeda, Merck, BMS, and receiving institutional research funding from AstraZeneca, Janssen, Merck, and Takeda. Dr. Sonam Puri reported having an institutional consulting or advisory role with Jazz Pharma, receiving consulting fees from G1 Therapeutics, Pfizer, Bristol-Myers Squibb, receiving honoraria from Aptitude Health, and support for travel, accommodations, and expenses from Dava Oncology. Dr. Stephen V. Liu reported having a consulting or advisory role with AbbVie, Amgen, AstraZeneca, Boehringer Ingelheim, Bristol Myers Squibb, Catalyst, Daiichi Sankyo, Eisai, Elevation Oncology, Genentech, Roche, Gilead Sciences, Guardant Health, Janssen Oncology, Jazz Pharmaceuticals, Merck, Merus, Mirati, Novartis, Regeneron, Sanofi, Takeda, and Turning Point Therapeutics, receiving institutional research funding from AbbVie, 10.13039/100018529Alkermes, Elevation Oncology, Ellipses, Genentech, Roche, 10.13039/100005564Gilead Sciences, Merck, Merus, Nuvalent, Inc., RAPT Therapeutics, and Turning Point Therapeutics, and participation in a data safety monitoring board or advisory board with Candel Therapeutics. Dr. Erminia Massarelli reported receiving honoraria from AstraZeneca, Merck, Eli Lilly and Company, Takeda Pharmaceuticals, Mirati, and having a consulting or advisory role with Bristol Myers Squibb, Daiichi Sankyo Co., Fusion Therapeutics, Janssen, Eli Lilly and Company, Sanofi, AbbVie, Iovance Therapeutics, and Mirati. Ms. Denise Williamson, Drs. Martin Schwickart, Christian Scheffold, and Svetlana Adrianova are employees of and reported having stock with Exelixis. In addition, Dr. Christian Scheffold reported a patent: Combinations of Cabozantinib and Atezolizumab to Treat Cancer; patent number 11198731. Dr. Enriqueta Felip reported having a consulting or advisory role with AbbVie, Amgen, AstraZeneca, Bayer, Boehringer Ingelheim, Bristol Myers Squibb, Eli Lilly, F. Hoffmann-La Roche, Gilead, GlaxoSmithKline, Janssen, Merck Serono, MSD, Novartis, Peptomyc, Pfizer, Regeneron, Sanofi, Takeda, Turning Point, Daiichi Sankyo, honoraria from Amgen, AstraZeneca, Bristol Myers Squibb, Daiichi Sankyo, Eli Lilly, F. Hoffmann-La Roche, Genentech, Janssen, Medical Trends, Medscape, Merck Serono, MSD, Peervoice, Pfizer, Sanofi, Takeda, Touch Oncology, and receiving support for travel, accommodations, and expenses from AstraZeneca, Janssen, Roche. The remaining authors declare no conflict of interest.
