# Supplementary material for: Cabozantinib Plus Atezolizumab or Cabozantinib Alone in Patients With Advanced NSCLC Previously Treated With an Immune Checkpoint Inhibitor: Results From the Phase 1b COSMIC-021 Study
Source: JTO Clin Res Rep. 2024 Mar 20;5(10):100666. doi: 10.1016/j.jtocrr.2024.100666 (PMC11421317; doi:10.1016/j.jtocrr.2024.100666)
Supplement: Supplementary Figure and Tables [file mmc1.docx]

**Supplementary Data**

**Cabozantinib Plus Atezolizumab or Cabozantinib Alone in Patients With Advanced Non-Small Cell Lung Cancer Previously Treated With an Immune Checkpoint Inhibitor: Results From the Phase 1b COSMIC-021 Study**

**Authors:** Joel W. Neal, Armando Santoro, Maria Gonzalez-Cao, Farah Louise Lim, Bruno Fang, Ryan D. Gentzler, Jerome Goldschmidt, Polina Khrizman, Claudia Proto, Shiven Patel, Sonam Puri, Stephen V. Liu, Erminia Massarelli, Denise Williamson, Martin Schwickart, Christian Scheffold, Svetlana Andrianova, Enriqueta Felip

**Table of Contents**

[**Supplementary Table 1.** Subsequent Therapy 2](#_Toc159319913)

[**Supplementary Table 2.** Tumor Response per RECIST v1.1 by BIRC 3](#_Toc159319914)

[**Supplementary Table 3.** Objective Response Rate, Overall Survival, Progression-Free Survival, and Duration of Response in Prespecified Subgroups 4](#_Toc159319915)

[**Supplementary Table 4.** Treatment Exposure 5](#_Toc159319916)

[**Supplementary Table 5.** Treatment-Emergent Adverse Events 6](#_Toc159319917)

[**Supplementary Table 6.** Adverse Events of Special Interest 8](#_Toc159319918)

[**Supplementary Figure 1.** Change in sum of target lesions over time per RECIST v1.1 by investigator in (A) combination cohort and (B) single-agent cabozantinib cohort. 9](#_Toc159319919)

[**Supplementary Figure 2.** Duration of therapy and response for (A) cabozantinib plus atezolizumab in combination cohort and (B) cabozantinib in single-agent cabozantinib cohort. 10](#_Toc159319920)

[**Supplementary Figure 3.** Overall survival in (A) combination cohort and (B) single-agent cabozantinib cohort. 11](#_Toc159319921)

[**Supplementary Figure 4.** Response, progression-free survival, and overall survival in combination cohort by tumor mutation burden. 12](#_Toc159319922)

[**Supplementary Figure 5.** Response, progression-free survival, and overall survival in combination cohort by presence of (A) *KRAS* mutation (B) *STK11* mutation and (C) *KEAP1 or STK11* mutation. 13](#_Toc159319923)

**Supplementary Table 1.** Subsequent Therapy

|  | **Cabozantinib + atezolizumab**  **(n=81)** | **Cabozantinib**  **(n=31)** |
| --- | --- | --- |
| Median time from enrollment to first subsequent systemic therapy, median (range), weeks | 23.1 (6.1–94.7) | 51.1 (23.1–77.0) |
| Any systemic therapy, n (%) | 24 (30) | 6 (19) |
| Protein kinase inhibitors, n (%) | 4 (5) | 2 (6) |
| Binimetinib | 1 (1) | 0 |
| Erlotinib | 1 (1) | 0 |
| Selpercatinib | 1 (1) | 0 |
| Naporafenib | 1 (1) | 0 |
| Trametinib | 1 (1) | 1 (3) |
| Cabozantinib | 0 | 1 (3) |
| Antibody-based therapies, n (%) | 9 (11) | 3 (10) |
| Pembrolizumab | 3 (4) | 0 |
| Bevacizumab | 2 (2) | 0 |
| Ramucirumab | 2 (2) | 1 (3) |
| Trastuzumab deruxtecan | 2 (2) | 0 |
| Durvalumab | 1 (2) | 0 |
| Atezolizumab | 0 | 1 (3) |
| GEN1046 | 0 | 1 (3) |
| Taxanes, n (%) | 12 (15) | 1 (3) |
| Docetaxel | 10 (12) | 1 (3) |
| Paclitaxel | 2 (2) | 0 |
| Pyrimidine analogues, (%) | 5 (6) | 1 (3) |
| Gemcitabine | 5 (6) | 1 (3) |

*Note:* Patients who crossed over from single-agent cabozantinib to cabozantinib plus atezolizumab were not counted as receiving subsequent therapy.

**Supplementary Table 2.** Tumor Response per RECIST v1.1 by BIRC

|  | **Cabozantinib + atezolizumab**  **(n=81)** | **Cabozantinib**  **(n=31)** |
| --- | --- | --- |
| ORR, % (95% CI) | 10 (4.4–18.5) | 6 (0.8–21.4) |
| Best overall response, n (%) |  |  |
| Confirmed complete response | 0 | 0 |
| Confirmed partial response | 8 (10) | 2 (6) |
| Stable disease | 61 (75) | 19 (61) |
| Progressive disease | 10 (12) | 5 (16) |
| Missing | 2 (2) | 5 (16) |
| Disease control rate, % (95% CI) | 85 (75.6–92.1) | 68 (48.6–83.3) |
| Duration of response, median (95% CI), months | 10.1 (4.7–NE) | 14.4 (NE–NE) |

*Note:* Disease control rate = complete response + partial response + stable disease ≥12 weeks.

BIRC, blinded independent radiology committee; CI; confidence interval; NE, not estimable; ORR, objective response rate; RECIST, Response Evaluation Criteria in Solid Tumors.

**Supplementary Table 3.** Objective Response Rate, Overall Survival, Progression-Free Survival, and Duration of Response in Prespecified Subgroups

|  | Cabozantinib + atezolizumab | | | | | | | | | | | | | |
| --- | --- | --- | --- | --- | --- | --- | --- | --- | --- | --- | --- | --- | --- | --- |
|  | Overall  (n=81) | ECOG PS | | PD-L1 status | | | | | Response to prior ICI^a^ | | | | Most recent therapy^b^ | |
|  |  | 0  (n=28) | 1  (n=52) | >1%–<49%  (n=18) | ≥50%  (n=19) | <1%  (n=37) | ≥1%  (n=40) | Unknown  (n=4) | CR+PR  (n=15) | SD  (n=35) | PD  (n=22) | CR+PR+SD  (n=50) | ICI^c^  (n=66) | Non-ICI^d^  (n=15) |
| ORR, %  (95% CI) | 20  (11.7–30.1) | 25  (10.7–44.9) | 17  (8.2–30.3) | 17  (3.6–41.4) | 26  (9.1–51.2) | 14  (4.5–28.8) | 23  (10.8–38.5) | 50  (6.8–93.2) | 20  (4.3–48.1) | 26  (12.5–43.3) | 18  (5.2–40.3) | 24  (13.1–38.2) | 21  (12.1–33.0) | 13  (1.7–40.5) |
| DOR, median (95% CI), months | 5.8  (4.2–6.9) | 4.9  (3.5–NE) | 6.7  (2.6–11.6) | 5.7  (4.2–NE) | NE  (6.9–NE) | 4.2  (2.6–NE) | 6.4  (3.5–NE) | 6.2  (5.8–NE) | 5.7  (4.2–NE) | 5.8  (2.6–6.7) | 6.9  (4.1–NE) | 5.8  (2.6–6.7) | 5.8  (4.2–6.9) | 7.57  (3.5–NE) |
| DCR, %  (95% CI) | 80  (69.9–88.3) | 79  (59.0–91.7) | 81  (67.5–90.4) | 78  (52.4–93.6) | 84  (60.4–96.6) | 78  (61.8–90.2) | 80  (64.4–90.9) | 100  (39.8–100.0) | 73  (44.9–92.2) | 80  (63.1–91.6) | 82  (59.7–94.8) | 78  (64.0–88.5) | 79  (67.0–87.9) | 87  (59.5–98.3) |
| PFS, median (95% CI), months | 4.5  (3.5–5.6) | 5.4  (2.8–6.7) | 4.1  (2.8–5.4) | 5.4  (2.1–7.2) | 5.4  (3.5–9.6) | 4.0  (2.7–5.6) | 4.8  (2.8–6.5) | 5.6  (4.0–NE) | 5.6  (1.4–8.3) | 4.1  (2.9–7.2) | 4.2  (2.6–5.4) | 5.4  (2.9–7.0) | 4.5  (3.5–5.7) | 4.5  (2.2–5.8) |
| OS, median (95% CI), months | 12.8  (7.2–15.7) | 16.3  (10.0–26.9) | 9.6  (6.0–14.5) | 15.8  (5.5–23.3) | 14.1  (5.9–NE) | 12.8  (5.4–15.7) | 13.3  (6.2–23.2) | 19.0  (4.6–NE) | 11.0  (2.2–15.7) | 14.1  (9.4–19.8) | 14.1 (4.9–17.1) | 12.7 (9.4–17.2) | 14.1  (9.6–17.4) | 6.0  (2.6–13.8) |

*Note:* Response outcomes and PFS were assessed by investigator.

^a^Single-agent ICI or ICI-containing regimen.

^b^Therapy received immediately prior to the enrollment.

^c^Single-agent ICI or ICI-containing regimen.

^d^Any therapy without ICI.

CI, confidence interval; CR, complete response; DCR, disease control rate; DOR, duration of response; ECOG PS, Eastern Cooperative Oncology Group performance status; ICI, immune checkpoint inhibitor; NE, not estimable; ORR, objective response rate; OS, overall survival; PD, progressive disease; PD-L1, programmed death-ligand 1; PFS, progression-free survival; PR, partial response; SD, stable disease.

**Supplementary Table 4.** Treatment Exposure

|  | **Cabozantinib + atezolizumab**  **(n=81)** | **Cabozantinib**  **(n=31)** |
| --- | --- | --- |
| Duration of exposure, median (range), months | 5.2 (0.3–30.2) | 3.5 (0.7–16.4) |
| Duration of exposure on cabozantinib and atezolizumab, median (range), months | 4.2 (0.0–29.7) | – |
| Any dose modifications (of any treatment component) due to AE, n (%)^a^ | 63 (78) | 27 (87) |
| Cabozantinib dose hold | 58 (72) | 25 (81) |
| Atezolizumab delay | 27 (33) | – |
| Cabozantinib dose reductions due to AE, n (%) | 34 (42) | 16 (52) |
| Reduction to 40 mg^b^ | – | 15 (48) |
| Reduction to 20 mg | 34 (42) | 7 (23) |
| Reduction to 20 mg every other day | 3 (4) | 1 (3) |
| Time to first dose reduction due to AE, median (range), days | 65.5 (9–413) | 52.5 (21–216) |
| Time to second dose reduction due to AE, median (range), days | 180.5 (127–239) | 102.5 (57–400) |
| TEAE leading to discontinuation, n (%) | 31 (38) | 6 (19) |
| Discontinuation of cabozantinib | 29 (36) | 6 (19) |
| Discontinuation of atezolizumab | 26 (32) | – |
| TRAE leading to discontinuation, n (%) | 14 (17) | 2 (6) |
| Discontinuation of cabozantinib | 12 (15) | 2 (6) |
| Discontinuation of atezolizumab | 9 (11) | – |

^a^Dose modification includes cabozantinib dose reductions due to AE, cabozantinib dose holds due to AE, and atezolizumab dose delays due to AE.
^b^The starting dose of cabozantinib was 40 mg for combination cohort.
AE, adverse event; TEAE, treatment-emergent adverse event; TRAE, treatment-related adverse event.

**Supplementary Table 5.** Treatment-Emergent Adverse Events

|  | **Cabozantinib + atezolizumab**  **(n=81)** | | **Cabozantinib**  **(n=31)** | |
| --- | --- | --- | --- | --- |
|  | **Any grade** | **Grade 3/4** | **Any grade** | **Grade 3/4** |
| Any event, n (%) | 81 (100) | 43 (53) | 31 (100) | 21 (68) |
| Diarrhea | 37 (46) | 1 (1) | 16 (52) | 3 (10) |
| Decreased appetite | 30 (37) | 1 (1) | 10 (32) | 1 (3) |
| Fatigue | 29 (36) | 4 (5) | 11 (35) | 2 (6) |
| Nausea | 29 (36) | 2 (2) | 14 (45) | 2 (6) |
| Asthenia | 25 (31) | 5 (6) | 12 (39) | 2 (6) |
| Constipation | 21 (26) | 0 | 4 (13) | 0 |
| Pyrexia | 20 (25) | 0 | 1 (3) | 0 |
| Aspartate aminotransferase increased | 19 (23) | 2 (2) | 9 (29) | 0 |
| Vomiting | 19 (23) | 0 | 9 (29) | 1 (3) |
| Hypertension | 18 (22) | 5 (6) | 10 (32) | 6 (19) |
| Alanine aminotransferase increased | 17 (21) | 3 (4) | 10 (32) | 1 (3) |
| Palmar-plantar erythrodysesthesia | 17 (21) | 3 (4) | 4 (13) | 0 |
| Hypomagnesemia | 16 (20) | 1 (1) | 5 (16) | 0 |
| Weight decreased | 16 (20) | 3 (4) | 4 (13) | 2 (6) |
| Anemia | 14 (17) | 2 (2) | 8 (26) | 1 (3) |
| Dyspnea | 14 (17) | 4 (5) | 6 (19) | 0 |
| Hyponatremia | 13 (16) | 3 (4) | 5 (16) | 2 (6) |
| Insomnia | 12 (15) | 0 | 1 (3) | 0 |
| Arthralgia | 11 (14) | 2 (2) | 3 (10) | 0 |
| Back pain | 11 (14) | 0 | 3 (10) | 1 (3) |
| Cough | 11 (14) | 0 | 1 (3) | 0 |
| Abdominal pain | 10 (12) | 1 (1) | 5 (16) | 0 |
| Headache | 10 (12) | 0 | 4 (13) | 0 |
| Hypothyroidism | 10 (12) | 0 | 6 (19) | 0 |
| Proteinuria | 10 (12) | 3 (4) | 4 (13) | 0 |
| Amylase increased | 9 (11) | 1 (1) | 2 (6) | 0 |
| Hyperglycemia | 9 (11) | 0 | 0 | 0 |
| Hypophosphatemia | 9 (11) | 2 (2) | 5 (16) | 1 (3) |
| Pneumonia | 9 (11) | 4 (5) | 4 (13) | 3 (10) |
| Rash | 9 (11) | 2 (2) | 2 (6) | 0 |
| Stomatitis | 7 (9) | 1 (1) | 7 (23) | 0 |
| Dysgeusia | 8 (10) | 0 | 5 (16) | 0 |
| Hypocalcemia | 8 (10) | 0 | 5 (16) | 0 |
| Urinary tract infection | 7 (9) | 1 (1) | 4 (13) | 0 |
| Hypokalemia | 6 (7) | 1 (1) | 4 (13) | 1 (3) |
| Blood creatinine increased | 5 (6) | 0 | 4 (13) | 0 |
| Dizziness | 4 (5) | 0 | 4 (13) | 0 |
| Productive cough | 4 (5) | 0 | 4 (13) | 0 |
| Thrombocytopenia | 4 (5) | 0 | 6 (19) | 0 |
| Gamma-glutamyltransferase increased | 3 (4) | 0 | 4 (13) | 0 |
| Fall | 1 (1) | 0 | 4 (13) | 1 (3) |

*Note*: This table reports treatment-emergent AEs that occurred in ≥10% of patients. Overall, 18 patients had a grade 5 treatment-emergent event in the combination cohort (disease progression [n=6], non-small cell lung cancer [n=5], lung adenocarcinoma [n=2], pneumonia [n=1], pneumonitis [n=1], completed suicide [n=1], listeria encephalitis [n=1], hemoptysis [n=1]) and 5 in the single-agent cabozantinib cohort (unspecified death [n=2], non-small cell lung cancer [n=1], lung adenocarcinoma [n=1], gastric ulcer hemorrhage [n=1]).

AE, adverse event.

**Supplementary Table 6.** Adverse Events of Special Interest

|  | **Cabozantinib + atezolizumab**  **Total (n=81)** | | **Cabozantinib**  **(n=31)** | |
| --- | --- | --- | --- | --- |
|  | **Any grade** | **Grade 3/4** | **Any grade** | **Grade 3/4** |
| Any AESI event, n (%) | 59 (73) | 21 (26) | 22 (71) | 3 (10) |
| Rash | 34 (42) | 7 (9) | 11 (35) | 1 (3) |
| Hepatitis (diagnosis and lab abnormalities) | 28 (35) | 6 (7) | 14 (45) | 1 (3) |
| Hepatitis (lab abnormalities) | 28 (35) | 5 (6) | 14 (45) | 1 (3) |
| Pancreatitis | 14 (17) | 5 (6) | 2 (6) | 0 |
| Hypothyroidism | 13 (16) | 0 | 7 (23) | 0 |
| Colitis | 3 (4) | 1 (1) | 1 (3) | 1 (3) |
| Hyperthyroidism | 3 (4) | 0 | 0 | 0 |
| Myocarditis | 1 (1) | 1 (1) | 0 | 0 |
| Ocular inflammatory toxicity | 1 (1) | 1 (1) | 0 | 0 |
| Pneumonitis | 3 (4) | 0 | 0 | 0 |
| Hepatitis (diagnosis) | 2 (2) | 1 (1) | 2 (6) | 0 |
| Infusion-related reactions | 2 (2) | 1 (1) | 0 | 0 |

*Note:* Adverse events of special interest are immune-mediated events associated with atezolizumab.

AESI, adverse event of special interest.

**Supplementary Figure 1.** Change in sum of target lesions over time per RECIST v1.1 by investigator in (A) combination cohort and (B) single-agent cabozantinib cohort.

Only patients with at least 1 baseline and post-baseline radiographic tumor assessment are shown.


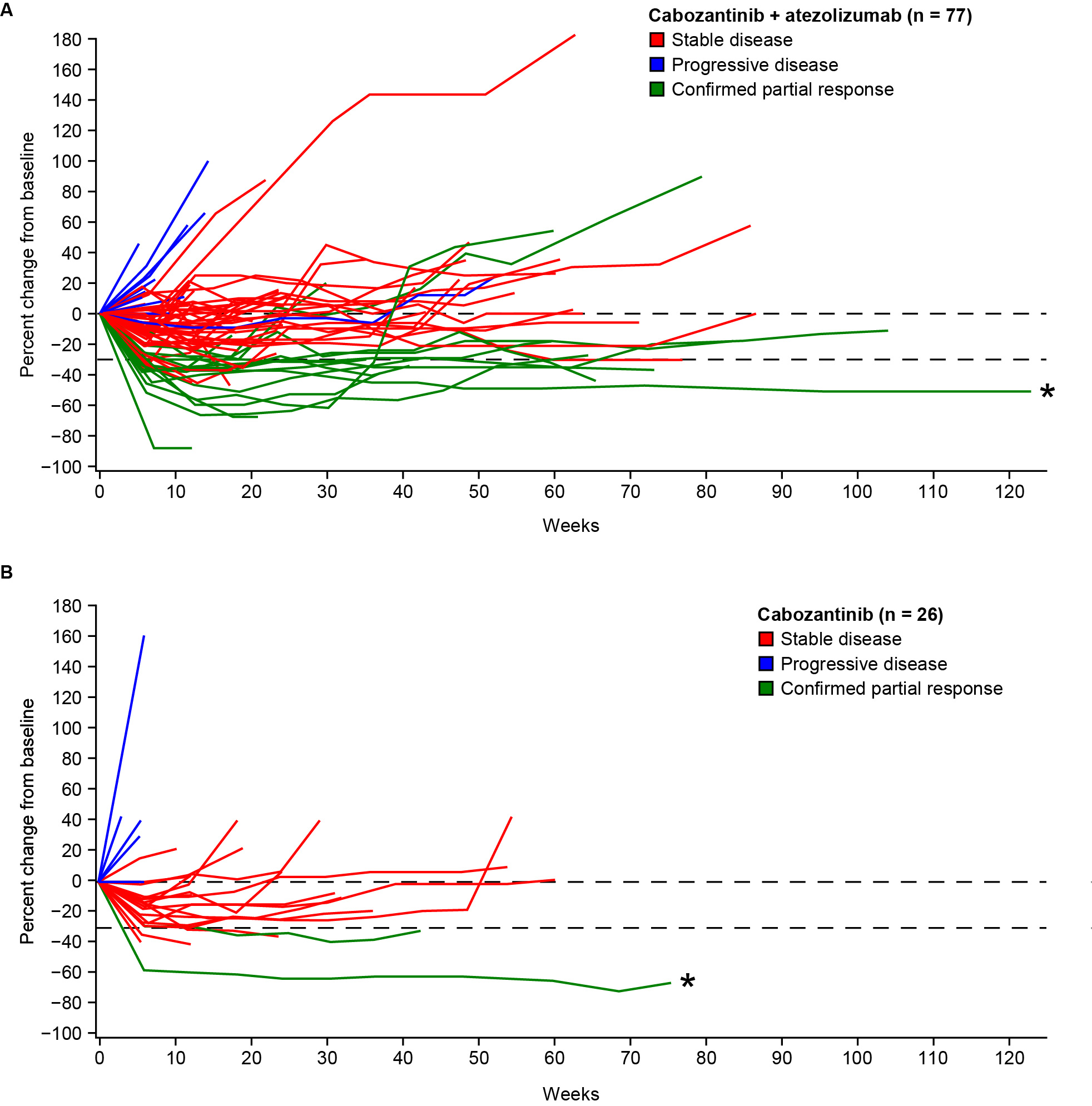


RECIST, Response Evaluation Criteria in Solid Tumors.

*Long-term response; both of these patients had no known genomic driver mutations, had brain metastases and had progressive disease as the best response with pembrolizumab-pemetrexed-carboplatin.

**Supplementary Figure 2.** Duration of therapy and response for (A) cabozantinib plus atezolizumab in combination cohort and (B) cabozantinib in single-agent cabozantinib cohort.


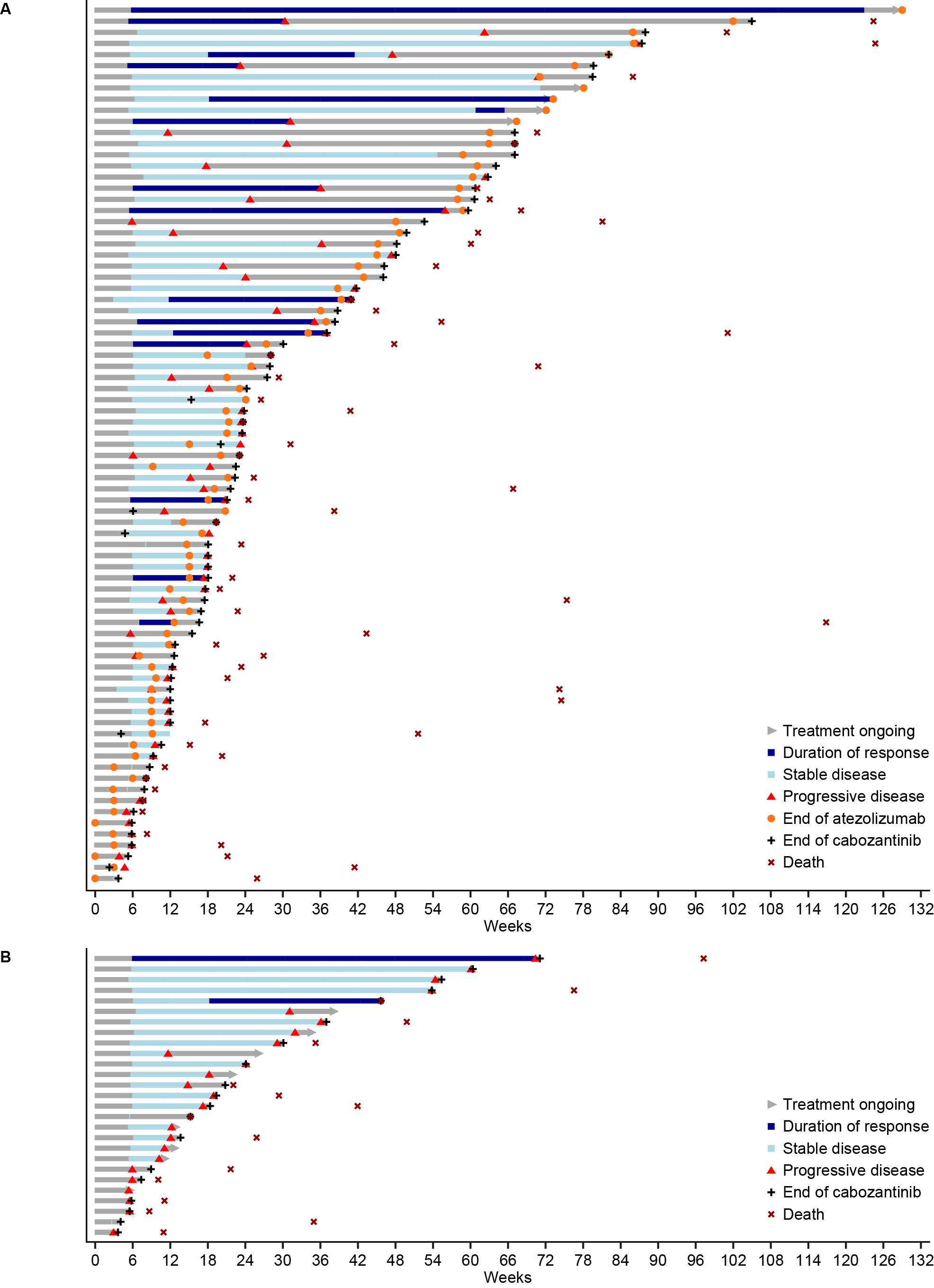


**Supplementary Figure 3.** Overall survival in (A) combination cohort and (B) single-agent cabozantinib cohort.


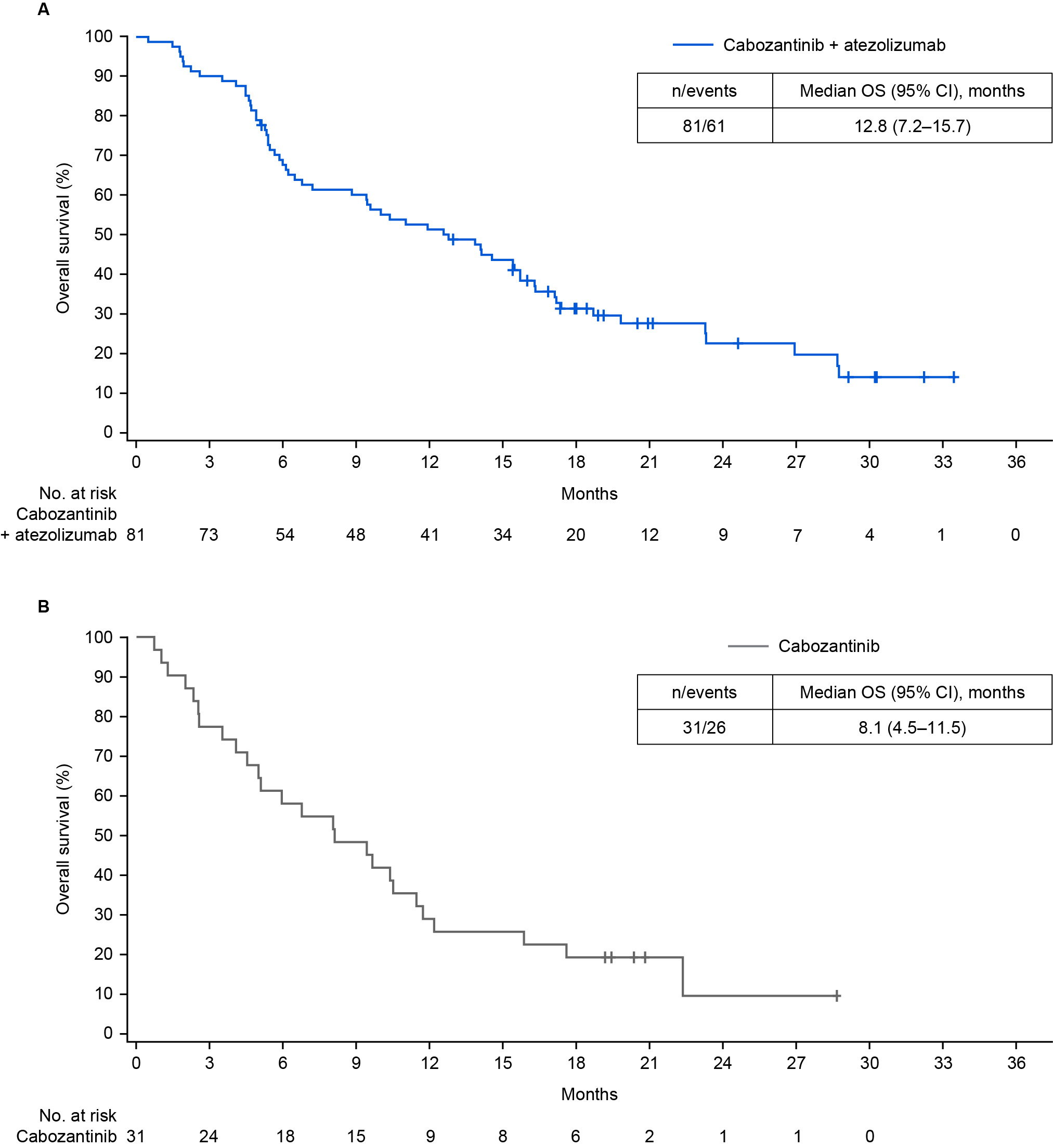


CI, confidence interval; No., number; OS, overall survival.

**Supplementary Figure 4.** Response, progression-free survival, and overall survival in combination cohort by tumor mutation burden.


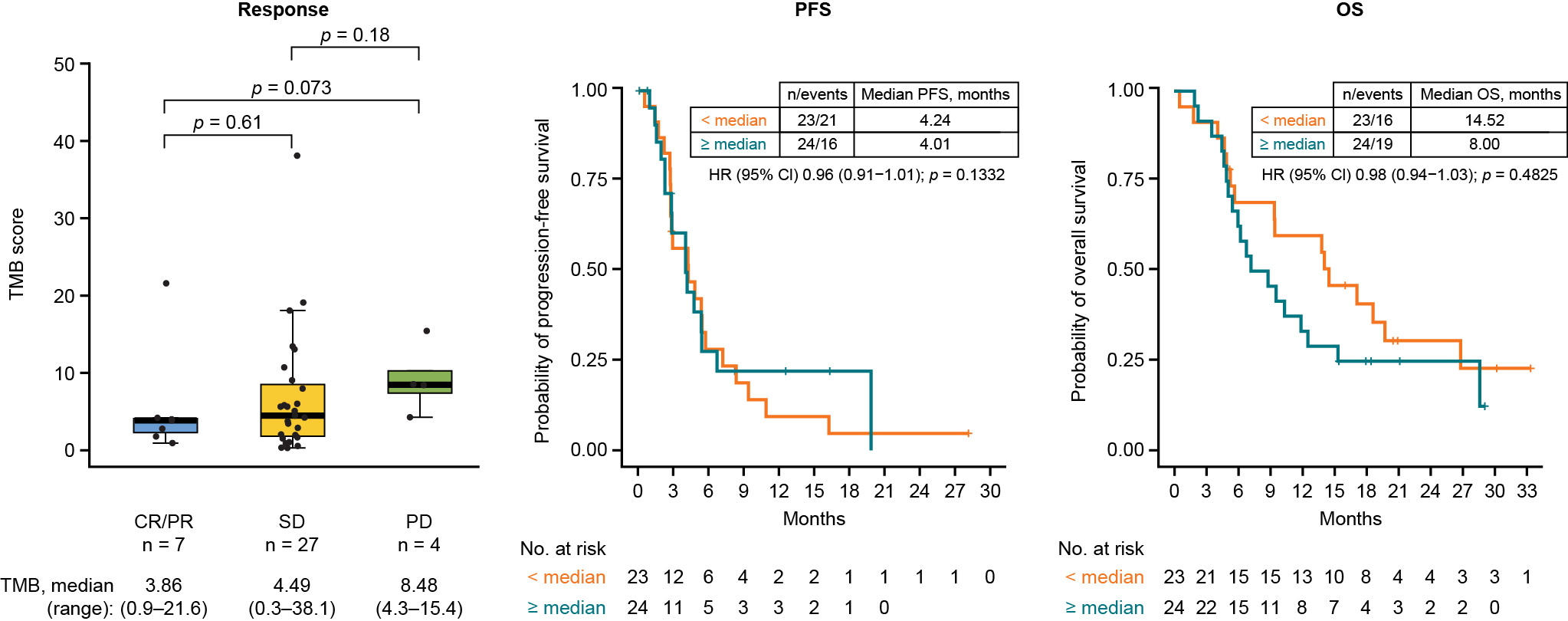


*Note:* In the Response plot, the line inside the box represents the median TMB score, and lower and upper boundaries of the boxes represent the 1st and 3rd quartiles, respectively; whiskers end at the highest data point within the third quartile plus 1.5 times interquartile range and lowest data point within the first quartile minus 1.5 times interquartile range; black circles represent individual TMB scores. Circles outside the error bars are outliers.

CI, confidence interval; CR, complete response; HR, hazard ratio; No., number; OS, overall survival; PD, progressive disease; PFS, progression-free survival; PR, partial response; SD, stable disease; TMB, tumor mutation burden.

**Supplementary Figure 5.** Response, progression-free survival, and overall survival in combination cohort by presence of (A) *KRAS* mutation (B) *STK11* mutation and (C) *KEAP1 or STK11* mutation.


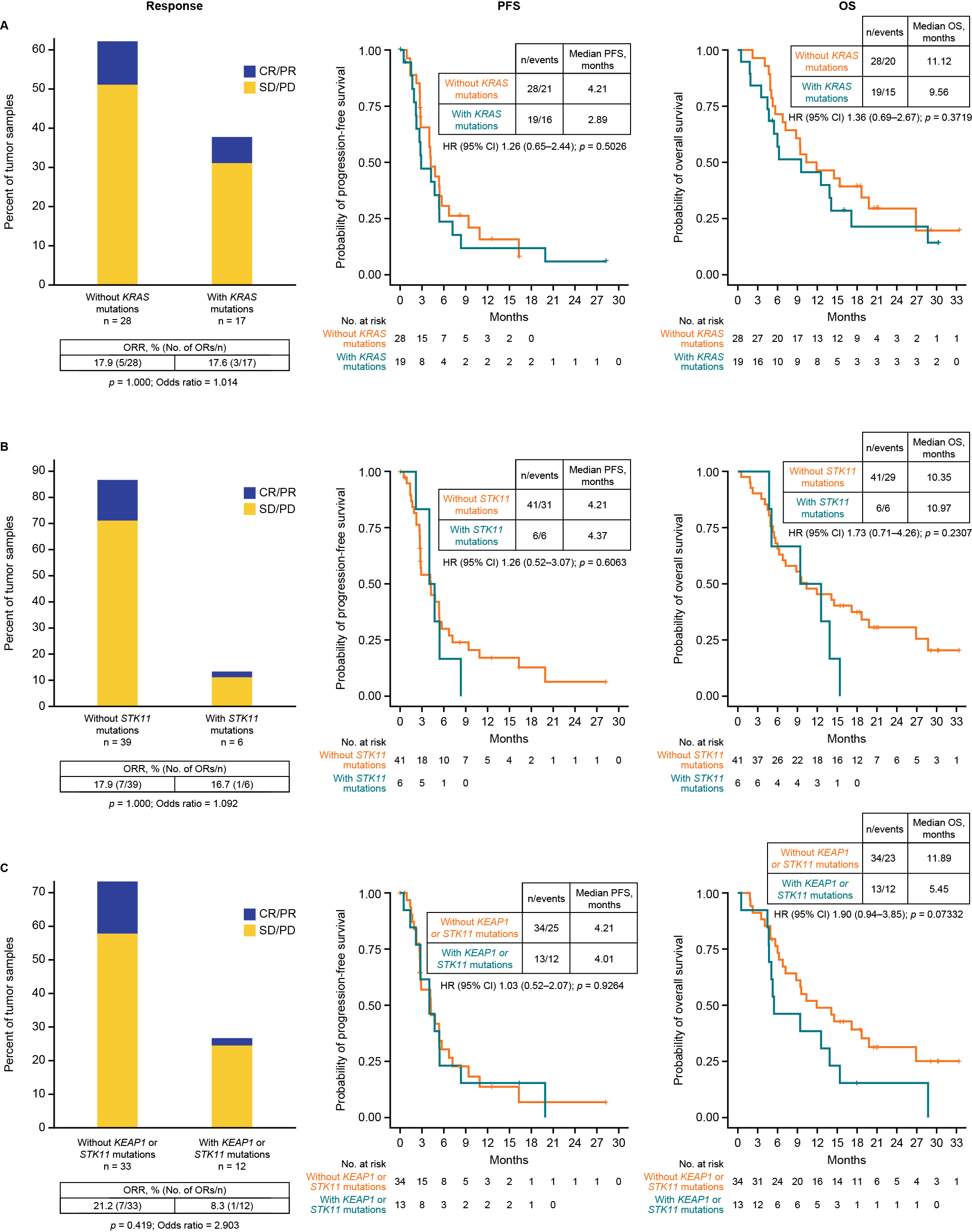


CI, confidence interval; CR, complete response; HR, hazard ratio; No., number; OR, objective response; ORR, objective response rate; OS, overall survival; PD, progressive disease; PFS, progression-free survival; PR, partial response; SD, stable disease.
